# Comparison of the accuracy of three intraocular lens power calculation formulas in cataract patients with prior radial keratotomy

**DOI:** 10.1186/s40001-023-00998-8

**Published:** 2023-01-11

**Authors:** Meng Li, Jin-Da Wang, Jing-Shang Zhang, Ying-Yan Mao, Kai Cao, Xiu-Hua Wan

**Affiliations:** Beijing Tongren Eye Center, Beijing Tongren Hospital, Capital Medical University, Beijing Ophthalmology & Visual Sciences Key Laboratory, 1 Dongjiaominxiang Street, Dongcheng District, Beijing, 100730 China

**Keywords:** Radial keratotomy, Cataract, Intraocular lens power calculation formula, Myopia, Absolute error

## Abstract

**Purpose:**

To compare the accuracy of three intraocular lens (IOL) formulas in Chinese cataract patients with prior radial keratotomy (RK).

**Methods:**

Medical records of cataract patients with prior RK at Beijing Tongren Hospital were retrospectively analysed. The absolute error (AE) was calculated as the absolute difference between the actual postoperative spherical equivalent and the predicted spherical equivalent. The AE and percentages of eyes with AE within 0.5D, 1.0D, and 2.0D for three formulas [Barrett True-K, Holladay 1 (D–K), Haigis] were calculated and compared.

**Results:**

Forty-seven eyes of 28 cataract patients were included. The Median AE (MedAE) was significantly different among the three formulas (*P* < 0.001). The MedAE was lowest for the Barrett True-K formula (0.62), followed by the Haigis (0.76), and Holladay 1 (D–K) (1.16). The percentages of eyes with AE within 0.5D, and 1.0D were significantly different among the 3 formulas (*P* = 0.009, and *P* < 0.001). The Barrett True-K formula achieved the highest percentages (46.8%) of eyes with AE within 0.5D. Haigis achieved the highest percentages (70.21%) of eyes with AE within 1.0 D.

**Conclusions:**

Barrett True-K is the most accurate IOL power calculation formula among the 3 formulas and Haigis is an alternative choice. Considering the relatively lower accuracy of IOL formulas in cataract patients with prior RK, newer and more accurate IOL formulas are desirable.

## Background

Myopia is the most common cause of distant visual impairment [1]. The prevalence of myopia has skyrocketed in the past few decades, especially in China [2]. Corneal refractive surgery is an effective way to correct adult myopia [3]. Radial keratotomy (RK), one of the corneal refractive surgeries, was developed in the 1980s [4]. Lots of people with myopia received RK surgery 30 years ago [4]. With the aging of these patients, more and more patients suffer from cataract and need cataract extraction and intraocular lens (IOL) implantation. Selecting a proper IOL with accurate power is an important factor in improving patient satisfaction [5]. Recently, several studies [6–13] compared the accuracy of newly developed IOL formulas in cataract patients with prior RK. However, there is no similar study from China which is a big country with myopia and myopia surgery. In addition, most Chines cataract patients with previous RK surgery lost their ocular data before RK surgery. Accurately calculating IOL power in Chinese cataract patients with prior RK and without previous ocular data remains a clinical challenge.

Barret True K with no history formula, a web-based IOL formula, was verified to be a good choice for cataract patients with prior RK surgery in several studies [7, 8, 10]. The official web of the American society of cataract and refractive surgeons provides several IOL formulas for cataract patients with previous corneal refractive surgery [14, 15]. If only IOL-Master data were available, Holladay 1 (Double-K) [Holladay 1 (D–K)] can be used to calculate IOL powers for cataract patients with prior RK. Haigis formula, a traditional formula, was also proved to be effective for cataract patients with prior RK surgery [6, 13]. In this study, we aimed to compare the accuracy of three IOL power calculation formulas in Chinese cataract patients with prior RK.

## Methods

### Patients

This study was performed in line with the principles of the Declaration of Helsinki. Approval was granted by the Ethics Committee of Beijing Tongren Hospital (Date: 2020-9-30/Number: TRECKY2020-069). Informed consent was obtained from all individual participants included in the study.

Consecutive cases that had previous RK surgery and had undergone a cataract surgery at Beijing Tongren Hospital between January 2011 and July 2020 were retrospectively reviewed. Inclusion criteria were patients with (1) previous RK surgery history, (2) available IOL-Master data taken before the cataract surgery, (3) no complications during or after the cataract surgery, and (4) manifest refraction performed between 1 and 6 months after the cataract surgery.

All cataract surgeries were performed by one surgeon (XH. W) using a clear corneal incision, phacoemulsification, and implantation of IOLs in the capsular bag according to our previous studies [16, 17].

### Intraocular lens power calculation formulas

Preoperative biometry was performed using the IOL-Master Biometer models 3, 500, or 700 (software 3.2, 7.5, and 1.50, respectively) (Carl Zeiss Meditec, Germany).

Three IOL power calculation formulas were used and analysed.

The Barrett True-K formula was developed for eyes with previous corneal refractive surgery [18]. In this study, the standard K was used with the Barrett True-K No History formula. The formula is available at http://www.apacrs.org/.

The Holladay 1 (D–K) formula was calculated on the official web of American society of cataract and refractive surgeons [14, 15]. The web address is http://www.ascrs.org/tools/post-refractive-iol-calculator.

Haigis formula was calculated using the software implemented in the IOL-Master Biometer.

### Prediction error and absolute error

The prediction error (PE) was calculated as the actual postoperative refraction minus the refractive result predicted by each formula. The absolute error (AE) was the absolute value of the PE.

The mean prediction error (MPE), mean absolute error (MAE), and median absolute error (MedAE) as well as the percentage of eyes that had an AE within 0.5, 1.0, and 2.0 D were calculated for each formula.

### Statistical analysis

Statistical analysis was performed using SPSS software version 20.0 (SPSS Inc., Chicago, IL, USA). Statistical analysis was performed according to Wang’s suggestion [19]. The 1-sample *t* test was used to determine whether the MPEs produced by various formulas were significantly different from zero. The differences in AE between formulas were analysed using the Friedman test. In the event of a significant result, post hoc analysis was undertaken using the Wilcoxon signed-rank tests for pairwise comparisons with Bonferroni correction. The differences in the percentage of eyes that had an AE within 0.5, 1.0, and 2.0 D between formulas were analysed using the Cochran Q test, post hoc analysis was undertaken using Dunn’s test for pairwise comparisons with Bonferroni correction. A *P* value of less than 0.05 was considered significant.

## Results

### Characteristics of the patients

A total of 47 eyes from 28 patients who had previous RK surgery and had undergone cataract surgery were recruited in this study. Of 28 patients, 18 were female and 10 were male. Among the 28 patients, 19 subjects received bilateral cataract surgery, and 9 subjects received unilateral surgery. Of 47 eyes, 25 were right eye and 22 were left eye. Further demographic details are shown in Table 1. IOLs implanted were 2 Tecnis ZMA00 (Abbott Medical Optics Inc, Santa Ana, CA, USA), 19 QUATRIX (CROMA-PHARMA GmbH, Leobendorf, Austria), 22 HOYA iSert 251 (HOYA co Ltd, Tokyo, Japan), and 4 Tecnis Symfony ZXR00 (Johnson & Johnson Vision, Santa Ana, CA, USA). The constants for the four IOLs are listed in Table 2 and were verified in lots of routine cataract patients in Beijing Tongren Hospital.

**Table 1:** Demographic details

	Mean or median	SD or range
Age (yrs)	54.39	7.53
RK incisions	12	6–16
Axial length (mm)	29.08	2.55
Mean K (D)	39.96	3.14
Anterior chamber depth (mm)	3.44	0.29
IOL power (D)	17.5	0–29

*SD* standard deviation, *D* diopters, *IOL* intraocular lens, *RK* radial keratotomy

**Table 2:** Intraocular lens calculation formula constants of different intraocular lens

Formula and constants	Tecnis ZMA00	CROMA QUATRIX	HOYA iSert 251	Symfony ZXR00
Barrett True-K: LF	2.15	2.2	1.62	2.09
Haigis: a0	− 1.750	1.91	− 0.542	− 0.1886
Haigis: a1	0.242	0.4	0.161	0.1716
Haigis: a2	0.266	0.1	0.204	0.1977
Holladay 1 (D–K): SF	2.06	2.28	1.52	1.96

*Holladay 1 (D–K)* Holladay 1 (Double-K)

### Mean predictive error and median absolute error

Detailed refractive outcomes for three intraocular lens calculation formulas are shown in Table 3. There was a small myopic bias in the MPE of the Barrette True K. The other two formulas showed a hyperopic bias of MPE. The MPEs of the three formulas were not significantly different from zero. Overall, the Barrette True-K formula showed the lowest MPE (− 0.235 ± 1.091 D). The highest MPE was found using the Holladay 1 (D–K) formula.

**Table 3:** Refractive accuracy for each intraocular lens calculation formula after previous radial keratotomy

Formula	MPE (95%CI)	SD of MPE	Range of PE	MAE	MedAE (25% to 75%)
Barrett True-K	− 0.235 (− 0.555 to 0.085)	1.091	− 2.440 to 3.920	0.813	0.620 (0.230 to 1.105)
Holladay 1 (D–K)	0.294 (− 0.154 to 0.742)	1.525	− 2.835 to 5.390	1.227	1.160 (0.535 to 1.605)
Haigis	0.246 (− 0.105 to 0.597)	1.196	− 2.290 to 4.580	0.905	0.760 (0.370 to 1.145)

*CI* confidence interval, *MPE* mean predictive error, *SD* standard deviation, *PE* predictive error, *MAE* mean absolute error, *MedAE* median absolute error, *Holladay 1 (D–K)* Holladay 1 (Double-K)

After Friedman test, the MedAEs of the three IOL formulas were significantly different (*X*^2^ = 18.553, *P* < 0.001). The Barrett True-K formula showed the lowest MedAE. After pairwise comparisons with Bonferroni correction, The Barrett True-K formula had significantly lower MedAE than the Holladay 1 (D–K) formula (*P* < 0.001). Likewise, the Haigis formulas had significantly lower MedAE than the Holladay 1 (D–K) formula (*P* = 0.001).

### Percentages of AE within 0.5 D, 1.0 D, and 2.0 D

**Fig. 1:**
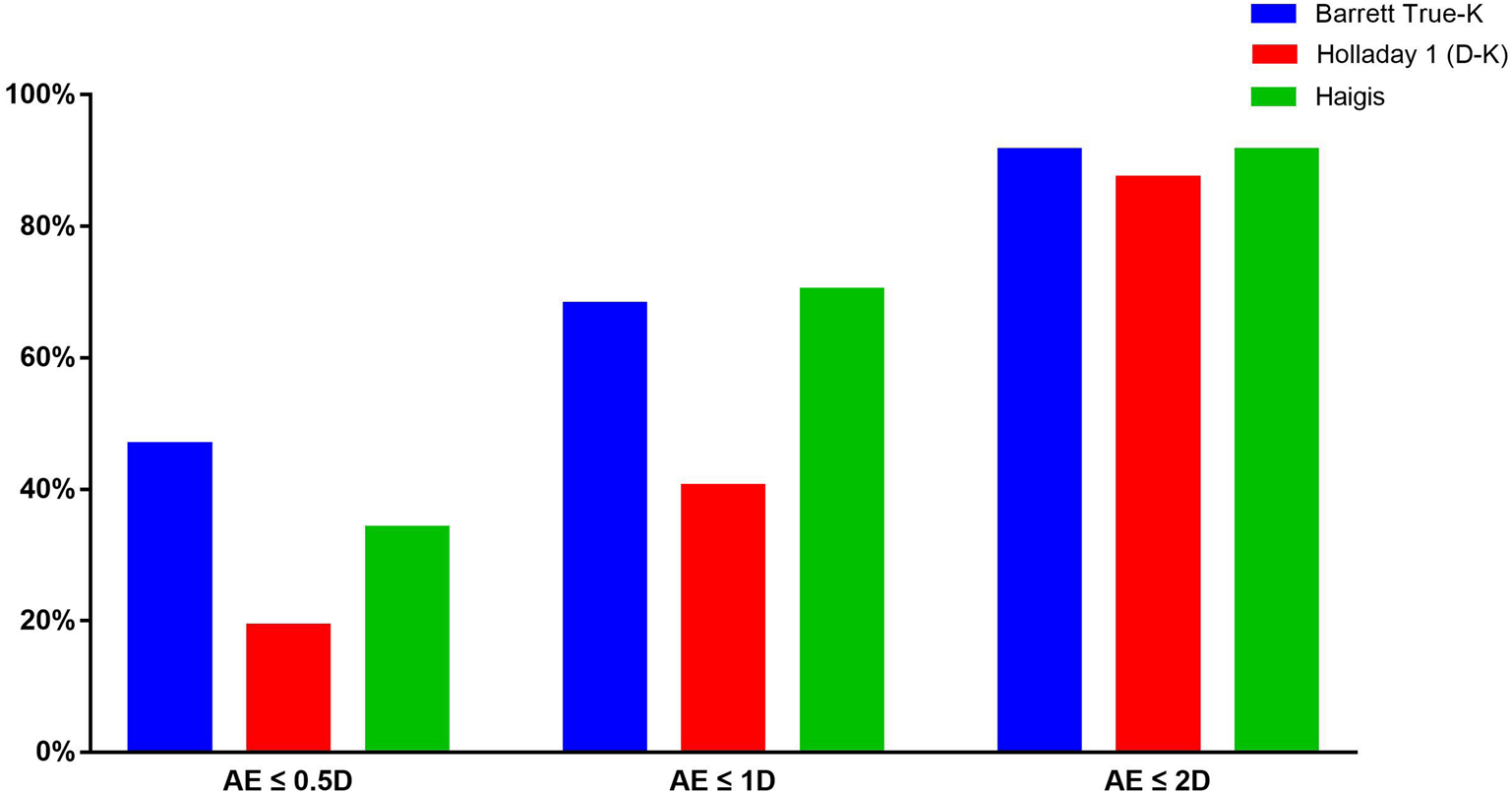
The percentage of eyes achieved the target absolute errors by the 3 formulas in cataract patients with prior radial keratotomy

The percentage of eyes that achieved the target AE by three formulas was displayed in Fig. 1.

The percentage of eyes with an AE within 0.5D was significantly different among the three formulas according to the Cochran Q test (*P* = 0.009). The Barrett True-K formula produced the highest proportion of eyes within 0.5D (46.81%), and the Holladay 1 (D–K) produced the lowest proportion (19.15%). Pairwise comparisons showed that the Barrett True-K formula produced a higher proportion of eyes within 0.5D than Holladay 1 (D–K) (*P* = 0.007).

The percentage of eyes with an AE within 1.0D was significantly different among the three formulas (*P* < 0.001). The Haigis formula produced the highest proportion of eyes within 1.0 D (70.21%), and the Holladay 1 (D–K) also produced the lowest proportion (40.43%). Pairwise comparisons showed that Haigis and Barrette True-K formulas produced a higher proportion of eyes within 1.0 D than Holladay 1 (D–K) (*P* = 0.001, and *P* = 0.003).

The percentage of eyes with an AE within 2.0D was not significantly different among the three formulas (*P* = 0.513). Both the Barrett True-K and Haigis formulas produced the highest proportion of eyes within 2.0 D (91.49%), and the Holladay 1 (D–K) also produced the lowest proportion (87.23%).

## Discussion

To our knowledge, this is the first study to assess the accuracy of three IOL power calculation formulas in a Chinese cohort with previous RK that underwent cataract surgery. Our study demonstrates that Barrett True-K is the most accurate IOL power calculation formula among the 3 formulas. In addition, Haigis is an alternative choice for these patients.

A recent study [20] from the UK assessed the accuracy of 3 new formulas and 6 established formulas in 10,930 eyes of 10,930 routine cataract patients and found that the AE of 68.1–72% eyes was within 0.5D. However, the accuracy of formulas in cataract patients who underwent prior RK is significantly lower. In our study, the most accurate formula is the Barrett True-K formula which achieved only 46.8% eyes with AE within 0.5D. The accuracy of IOL power formulas in RK patients is poor for the following reasons. First, RK surgery not only changes the anterior corneal curvature, but also the posterior corneal curvature [21]. Most formulas used a fixed ratio to determine the posterior corneal refractive power according to the anterior corneal curvature, which leads to overestimating the corneal power and results in postoperative hypermetropia [22]. In our study, both Holladay 1 (D–K) and Haigis formulas showed a hyperopic bias. Second, most IOL power formulas predicted the effective lens position according to the corneal power [23]. After RK surgery, the corneal power changed significantly, which resulted in an inaccurate effective lens position estimation and postoperative hypermetropia for most IOL power formulas [22].

In this study, the Barrett True-K formula achieved the lowest MedAE and the highest percentage of eyes with AE within 0.5D among 3 formulas in cataract patients with prior RK. Several previous studies [6–12] evaluated the accuracy of the Barrett True-K formula in cataract patients with prior RK (Table 4). Curado’s study [10] showed a similar result to our study. Curado’s study [10] compared the accuracy of 7 IOL power formulas in 52 eyes of 34 patients with prior RK surgery and found that the Barrett True-K formula was superior to the other 6 formulas (ORA, SRK/T, Hoffer Q, Haigis, Holladay 1, and Holladay 2). Another study [8] from Australia not only showed that the Barrett True-K formula was superior to other formulas in cataract patients with prior RK surgery but also found that the more the patient’s prior medical data, the higher the accuracy of the Barrett True-K formula. In that study, the percentages of eyes with AE within 0.5D were 76.6% using the Barrett True-K formula with complete previous medical data, 75.0% using the Barrett True-K formula with partial previous medical data, and 69.2% eyes using the Barrett True-K formula with no previous medical data. In our study, we only evaluated one Barrett True-K formula due to incomplete previous medical data of patients with prior RK surgery. Dawson’s study [7] showed that the Barrett True-K formula performed similar to our study. In that study, the Barrett True-K formula achieved 51.1% eyes with AE within 0.5D [7]. However, there are still few studies [9, 11] that showed the Barrett True-K formula was not accurate in patients with prior RK. Patel’s study [9] showed that the percentage of eyes with AE within 0.5D for the Barrett True-K formula was only 22.22%, which was significantly lower than our study. The difference in percentages of eyes with AE within 0.5D for the Barrett True-K formula in different studies may be due to different ocular biometer parameters, different postoperative visit times, and different sample sizes in different studies.

**Table 4:** Summaries of recent studies that assessed the IOL power calculation formulas in cataract patients with prior radial keratotomy

Author	country	Sample	MedAE	AE ≤ 0.5D	AE ≤ 1D
Dawson [8]	USA	47 eyes (31 patients)	True-K (0.50), intraoperative aberrometry (0.48)	True-K (51.1%), intraoperative aberrometry (55.3%)	True-K (80.8%), intraoperative aberrometry (80.8%)
Leite [13]	Brazil	108 eyes	NR	Haigis (43.5%), True-K (42.6%)	Haigis (65.7%), True-K (75.9%)
Turnbull [9]	Australia	52 eyes (34 patients)	TKH (0.275), TKPH (0.382), TKNH (0.330), Haigis (0.406), Holladay (0.504), Haigis [-0.50 offset] (0.569), Potvin-Hill (0.632)	TKH (76.6%), TKPH (75.0%), TKNH (69.2%), Haigis (69.2%), Holladay (50.0%), Haigis [-0.50 offset] (46.2%), Potvin-Hill (40.4%)	TKH (97.9%), TKPH (95.8%), TKNH (92.3%), Haigis (92.3%), Holladay (84.6%), Haigis [-0.50 offset] (82.7%), Potvin-Hill (69.2%)
Patel [10]	India	54 eyes (36 patients)	NR	Atlas 1–4 (19.44%), IOLMaster (22.22%), True-K (22.22%), average IOL power (27.78%), minimum IOL power (22.22%), maximum IOL power (22.22%)	Atlas 1–4 (47.22%), IOLMaster (50.00%), True-K (50.00%), average IOL power (58.33%), minimum IOL power (52.78%), maximum IOL power (50.00%)
Curado [11]	Brazil	52 eyes (34 patients)	True-K (0.34), ORA (0.53), SRK/T (0.54), Hoffer Q (0.51), Haigis (0.54), Holladay 1 (0.57), Holladay 2 (0.44)	True-K (63.5%), ORA (48.1%), SRK/T (44.2%), Hoffer Q (48.1%), Haigis (53.8%), Holladay 1 (36.5%), Holladay 2 (57.7%)	True-K (88.5%), ORA (80.7%), SRK/T (76.9%), Hoffer Q (84.6%), Haigis (75.0%), Holladay 1 (76.9%), Holladay 2 (84.6%)
Wang [14]	USA	44 eyes (32 patients)	Haigis (0.47), True-K (0.55), Haigis-TK (0.57)	Haigis (54.5%), True-K (43.2%), Haigis-TK (43.2%)	Haigis (75.0%), True-K (70.5%), Haigis-TK (72.7%)
This study	China	47 eyes (28 patients)	True-K (0.62), Holladay 1 (D–K) (1.16), Haigis (0.76)	True-K (46.8%), Holladay 1 (D–K) (19.1%), Haigis (34.0%)	True-K (68.1%), Holladay 1 (D–K) (40.4%), Haigis (70.2%)

*IOL* intraocular lens, *MedAE* median absolute error, *NR* not report, *TKH* True-K [History], *TKPH* True-K [partial history], *TKNH* True-K [no history], *ORA* the optiwave refractive analysis, *Holladay 1 (D–K)* Holladay 1 (Double-K)

In this study, we also found that the Haigis formula, a traditional formula, was an alternative choice for cataract patients with prior RK surgery. In our study, the Haigis formula achieved the highest percentage of eyes with AE within 1.0D and the same percentage of eyes with AE within 2.0D as the Barrett True-K formula. Two previous studies [6, 13] showed that the Haigis formula was superior to other formulas in predicting IOL power for cataract patients with prior RK surgery. Wang’s study [13] even verified that Haigis was superior to the Barrett True-K formula. Haigis achieved 54.5% eyes with AE within 0.5D, while Barrett True-K only achieved 43.2%. The relatively good performance of the Haigis formula in cataract patients with prior RK may be due to the following reasons. First, the Haigis formula predicted effective lens position without taking into account the corneal power which was significantly changed after RK surgery [13]. Second, previous studies [24, 25] showed that the Haigis formula achieved good performance in cataract patients with long axial length. In our study, the mean axial length of cataract patients with prior RK surgery was 29.08 ± 2.55 mm, which was significantly longer than that of normal cataract patients.

In this study, Holladay 1 (D–K) only achieved 19.15% eyes with AE within 0.5D. The poor performance of Holladay 1 (D–K) formulas may be due to the following reason. In this study, all patients lost their ocular data before RK surgery, no prior ocular data were imputed into the calculator. In addition, only parts of the patients received corneal tomography examination before cataract surgery, corneal tomography data were also not used. In this study, only IOL-Master data were imputed into the calculator, which may lead to the poor performance of Holladay 1 (D–K) formulas.

The accuracy of IOL power formulas in cataract patients with prior RK surgery was lower than that in normal cataract patients. Even for the most accurate formula, the Barrett True-K formula, less than 50% eyes with AE within 0.5D was achieved in our study. With the accumulation of similar cases, improvement of ocular biometry measurements [26], application of artificial intelligence, and big data technology [27], more accurate IOL formulas for cataract patients with prior RK surgery may be developed.

There are several limitations to this study. First, the time of postoperative manifest refraction examination was not uniform for every patient due to the retrospective nature of this study. Second, both eyes of some patients were included in this study, the between-eye correlation may affect our results. Third, most patients lost their medical data of RK surgery, and newer IOL formulas that incorporate patients’ prior refractive data were not compared in our study.

In conclusion, the Barrett True-K formula was the most accurate formula for cataract patients with prior RK in this Chinese cohort. The Haigis formula was an alternative choice for these patients. Despite this, the results in cataract patients with prior RK remain significantly less accurate compared with routine cataract patients, indicating the need for further research to improve the accuracy of IOL formulas in eyes with prior RK.
